# Possible Transformation of Pseudotumor to Synovial Sarcoma in a Failed Metal-on-Metal Total Hip Arthroplasty

**DOI:** 10.1016/j.artd.2024.101408

**Published:** 2024-09-26

**Authors:** Victor Shen, Reed Andrews, Odion Binitie, Brian T. Palumbo

**Affiliations:** a Department of Orthopaedic Surgery, University of Southern Florida Health Morsani College of Medicine, Tampa, FL, USA; b Department of Orthopaedic Surgery, Florida Orthopaedic Institute, Tampa, FL, USA; c Department of Orthopaedic Surgery, Moffitt Cancer Center, Tampa, FL, USA

**Keywords:** Pseudotumor, Synovial sarcoma, Metal-on-metal, Total hip arthroplasty

## Abstract

Adverse local tissue reaction (ALTR) from the release of chromium and cobalt ions in metal-on-metal total hip arthroplasty (MoM THA) is a well-reported complication, but there is little evidence suggesting that this inflammatory reaction causes malignancy. We present a 69-year-old female with MoM THA who developed synovial sarcoma (SS). She underwent mass resection and revision THA. Postoperative pathologic analysis revealed the unanticipated diagnosis of SS. She subsequently underwent chemotherapy, sarcoma resection, and endoprosthetic reconstruction. We hypothesize that the SS developed from an ALTR in the setting of failed MoM THA. Given the paucity of data on possible malignant transformation of an ALTR to SS, we advise surgeons to consider potential malignancies when diagnosing ALTR in the setting of failed MoM THA bearings.

## Introduction

Metal-on-metal (MoM) bearing surfaces in total hip arthroplasty (THA) were first popularized in the 1960s due to their potential for low wear rates and minimal osteolysis. [1] However, increasing reports of metal particle-induced type IV hypersensitivity reactions or aseptic lymphocyte-dominant vasculitis-associated lesions led to the decline of these bearings. [2,3] These lesions are referred to as adverse local tissue reactions (ALTRs) or pseudotumors, given their resemblance to sarcomas on magnetic resonance imaging (MRI) and clinical examination. Current treatment includes resection or debulking with revision of the bearing to a ceramic-on-polyethylene articulation. [4,5] As many as one-third of patients with MoM THA have reported radiographic evidence of an ALTR. [4,6]

Excess production of metallic particulate debris, which includes chromium (Cr) and cobalt (Co) ions from “at-risk” or malpositioned implants may increase the potential for associated pathology. [[7], [8], [9], [10], [11]] Contemporary MoM THA bearings produce 13,500 times more Co/Cr ions than metal-on-polyethylene bearings. [12] These ions have been shown to be possible carcinogens in vitro. [13,14] Though host inflammatory reactions to Co/Cr ions have been associated with the development of malignancy, there is little evidence to suggest in vivo carcinogenesis in orthopaedic patients. [12,[15], [16], [17]]

Cases of B-cell lymphoma, angiosarcoma, and renal clear cell carcinoma in patients with MoM THA have been reported. [18,19] In 1988, Martin et al. reported a case of telangiectatic sarcoma associated with a THA. [20] Herein, we describe the case of a patient with MoM THA who underwent revision for presumed ALTR with intraoperative pathology demonstrating synovial sarcoma (SS).

## Case history

**Figure 1:**
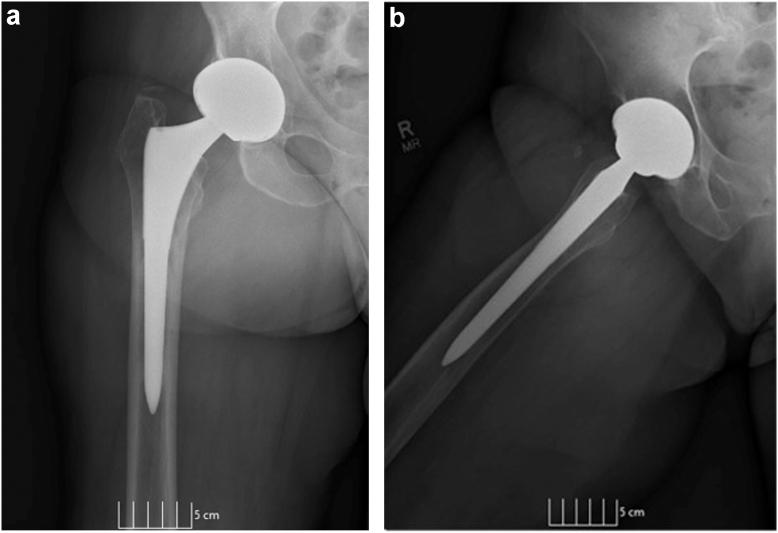
(a) Anterior-posterior (AP) and (b) frog lateral radiographs of right hip showing metal-on-metal total hip replacement without obvious evidence of peri-implant osteolysis or other acute changes. Soft tissue swelling was noted in the inguinal region.

**Figure 2:**
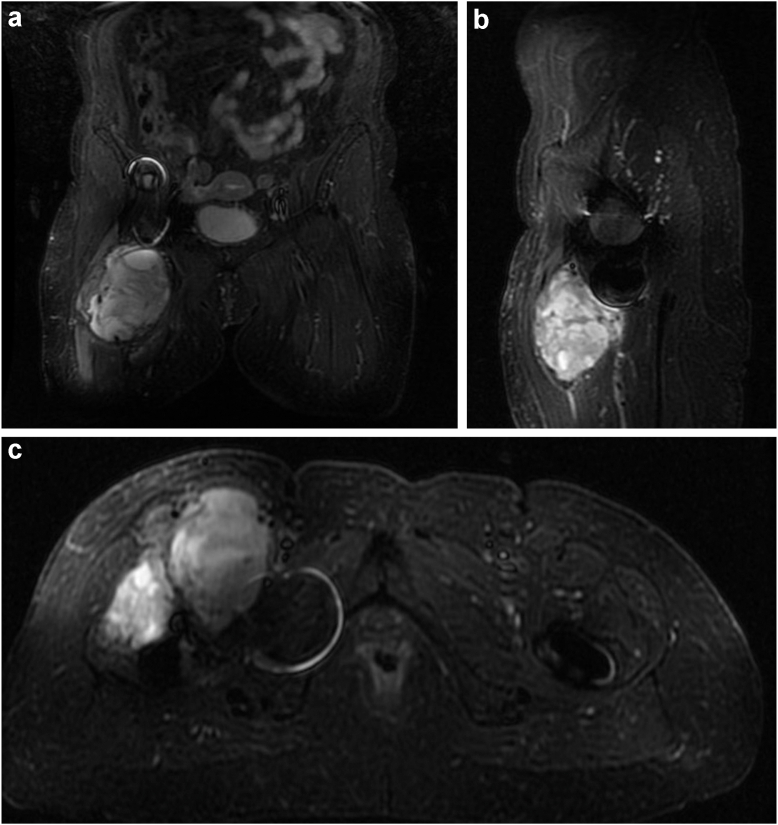
(a) Coronal, (b) sagittal, and (c) axial MRI images demonstrating a lobular, irregular, large mass anterior to the proximal femoral shaft with high signal intensity on fluid-weighted sequencing.

A 69-year-old female presented to our clinic in 2016 with a painful right MoM THA (Depuy, Summit stem and Pinnacle Acetabular socket). This stem has a single modular head-neck junction and cobalt-chromium head. The socket is made of titanium, a modular cobalt-chromium liner. Several years before her right THA, she had undergone a successful left THA with a ceramic-on-polyethylene articulation. She presented to our clinic with a 1-year history of worsening and constant groin pain, an antalgic gait, and a notable mass in the upper thigh and groin. She complained of her inability to ambulate long distances and carry out regular activities of daily living. On physical exam, range of motion was mildly painful and consisted of 90° hip flexion, 20° adduction, 45° abduction, and limited internal rotation of 0°. Pain was elicited with flexion, adduction, and internal rotation, and she had a positive Stinchfield test. She was neurovascularly intact throughout all peripheral motor and sensory distributions of the right lower extremity. Weight-bearing anterior-posterior and lateral radiographs of the right hip (See Fig. 1) demonstrated a well-positioned MoM THA with well-fixed components in acceptable alignment. A metal artifact reduction sequence (MARS) MRI demonstrated a lobular irregular large mass anterior to proximal femoral shaft. (See Fig. 2) Serologic analysis revealed elevated serum Co and Cr ions of 3.4 μg/L and 1.0 μg/L, respectively. A hip aspiration performed by the referring physician was sent to a community pathologist, who read the specimen as positive for clusters of epithelioid small round cells. The pathology results and magnetic resonance findings were reviewed and discussed with a musculoskeletal oncology attending. While a soft tissue neoplasm was considered, the appearance of an ALTR varies widely on MRI and histology. Histological analysis of ALTR has shown that it contains macrophages aggregating around metal particles. [21,22] Although aseptic lymphocyte-dominant vasculitis-associated lesions is lymphocytic infiltrate predominant, patients with higher wear have been shown to have fewer lymphocytes and more macrophages [23]. Given the context of the close vicinity of the mass to a MoM bearing, elevated cobalt ion levels, and a lack of literature reporting neoplastic development secondary to MoM THA, a presumptive diagnosis of right hip ALTR secondary to failed MoM was made. The initial treatment plan was to debulk the ALTR mass, send tissue for permanent pathologic analysis, and replace the bearing with a ceramic on a highly crosslinked polyethylene articulation.

**Figure 3:**
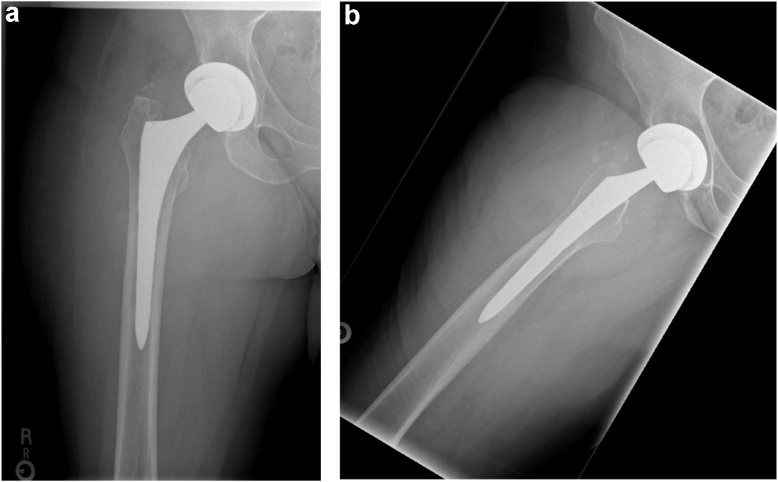
(a) Anterior-posterior (AP) and (b) frog leg lateral radiographs of the right hip 1-month status postrevision to ceramic-on-polyethylene bearing.

A posterolateral approach was performed. Upon capsulotomy, excess black fluid consistent with component impingement and metal debris was expressed from the joint. Cultures were obtained, and tissue was sent for frozen analysis, which was negative for acute inflammation but notable for “focal chronic synovitis.” No significant neutrophilic infiltrate was found. During the approach, a sinus or soft tissue tunnel was noted to communicate from the hip joint, anterior to the gluteus medius, directly into the anterior thigh, and connecting to the ALTR. The femoral head and stem revealed severe staining with black residue at both male and female ends, consistent with trunnionosis and mechanically assisted crevice corrosion. During debulking and resection of the mass, abundant perforating vessels were encountered, and excessive bleeding commenced. As the tumor was resected, bleeding became more intense and blood loss escalated rapidly to approximately 1600 mL. Given the atypical and excessive nature of the bleeding, we aborted further mass resection, and adequate hemostasis was obtained via electrocauterization, suture ligation, and thrombolytic agents. The modular metal components were replaced with ceramic-on-polyethylene bearing, and the hip was closed. (See Fig. 3)

**Figure 4:**
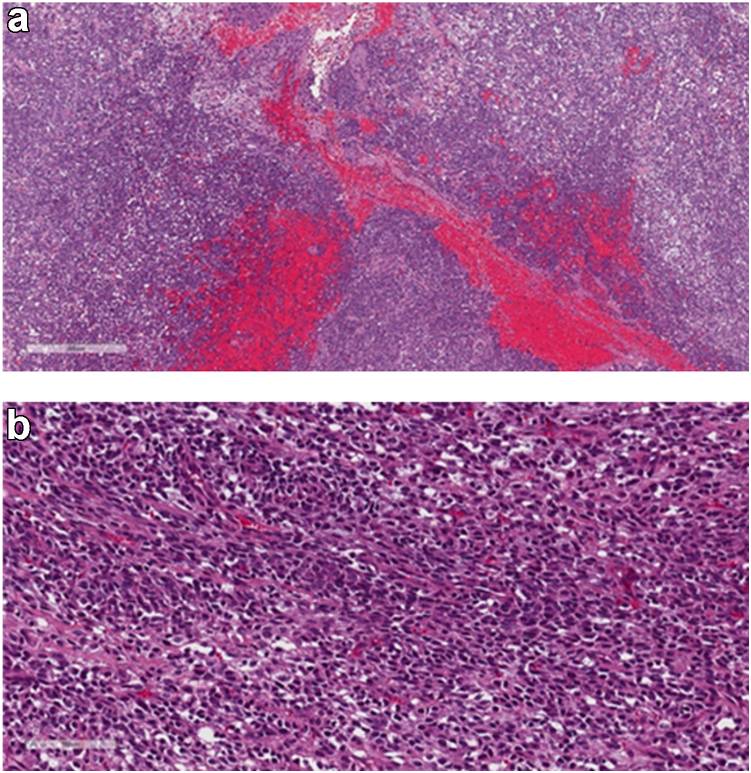
(a) Low-power view and (b) higher-power view of hematoxylin and eosin-stained slides show the tumor is composed of hypercellular spindle cells with monotonous appearance. There is scant cytoplasm. The nuclei are hyperchromatic. The tumor is immunoreactive to vimentin, CD99, BCL2, and TLE1, not shown here. SS18 (SYT) rearrangement is detected by fluorescence in situ hybridization, not shown here.

Specimens of the mass were sent for permanent section analysis, which later returned as being consistent with a poorly differentiated, high-grade Fédération Nationale des Centres de Lutte Contre le Cancer grade III SS. Immunohistochemical staining was positive for TLE-1, epithelial membrane antigen, Bcl-2, vimentin, and Ki-67 (10%-20%). Fluorescence in situ hybridization (FISH) analysis was positive for the SS18 (SYT) gene rearrangement. (See Fig. 4) A t(4;18) chromosomal translocation was revealed by FISH analysis. Because of the unusual clinical presentation of our case, the tissue specimen was sent to the University of Miami for repeat analysis confirming our histologic assessment.

**Figure 5:**
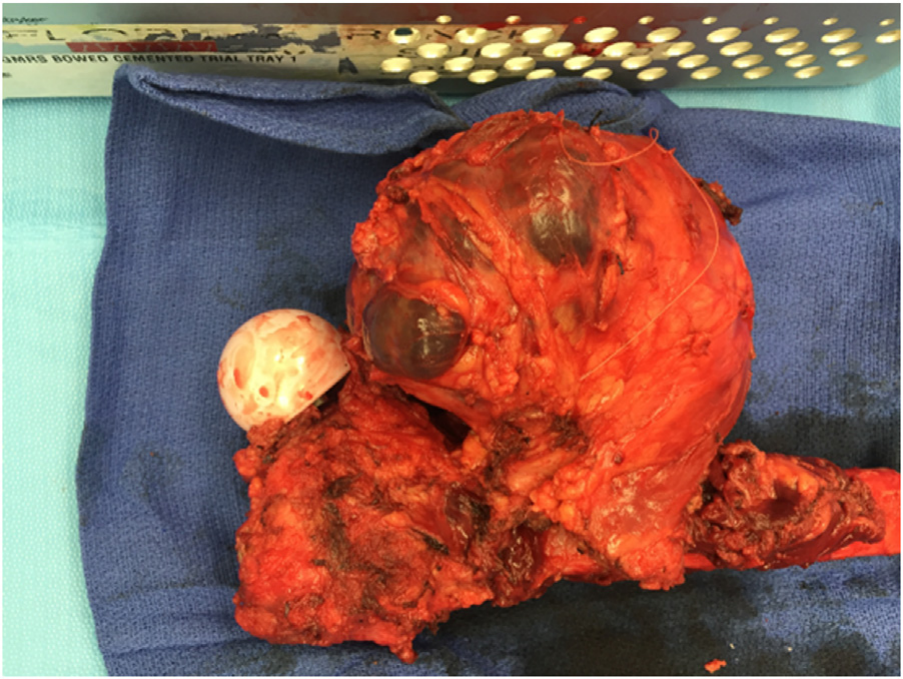
Gross specimen after resection of the proximal femur, SS tumor, and connecting sinus.

**Figure 6:**
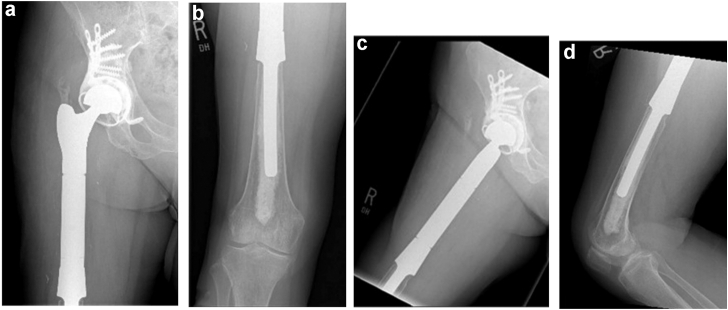
(a) Right hip anterior posterior (AP), (b) right distal femur AP, (c) right hip frog leg lateral, and (d) right distal femur lateral radiographs 5-year status postproximal femur replacement and acetabular reconstruction.

After diagnosis, the patient was transferred to a regional tertiary referral cancer center, where a multidisciplinary sarcoma team assumed her care. She was first appropriately staged and found not to have metastatic disease. The treatment plan was determined to include 2 cycles of neoadjuvant chemotherapy consisting of doxorubicine and ifosfamide, followed by surgery and radiation. After the completion of chemotherapy, she was restaged and still showed no signs of metastatic disease. However, a slight increase in tumor size was noted on the MRI. After completing neoadjuvant chemotherapy and 4 months after the hip revision, she underwent wide surgical resection and reconstruction through a posterolateral approach. An en bloc resection of the previous scar, proximal femur, sarcoma, and the communicating tract connecting the joint to the sarcoma was performed. (See Fig. 5) Subsequently, a proximal femoral reconstruction was performed with a Global Modular Replacement System endoprosthesis and acetabular reconstruction using a cup-cage construct and tripolar constrained bearing (Stryker, Mahwah, NJ). (See Fig. 6) She had an uncomplicated early recovery and underwent 64-Gy of adjuvant radiation. Afterward, she regained good function, was ambulating with a cane, and transitioned to surveillance monitoring. However, 6 months later, a pulmonary computed tomography demonstrated several subcentimeter nodules concerning metastatic disease.

**Figure 7:**
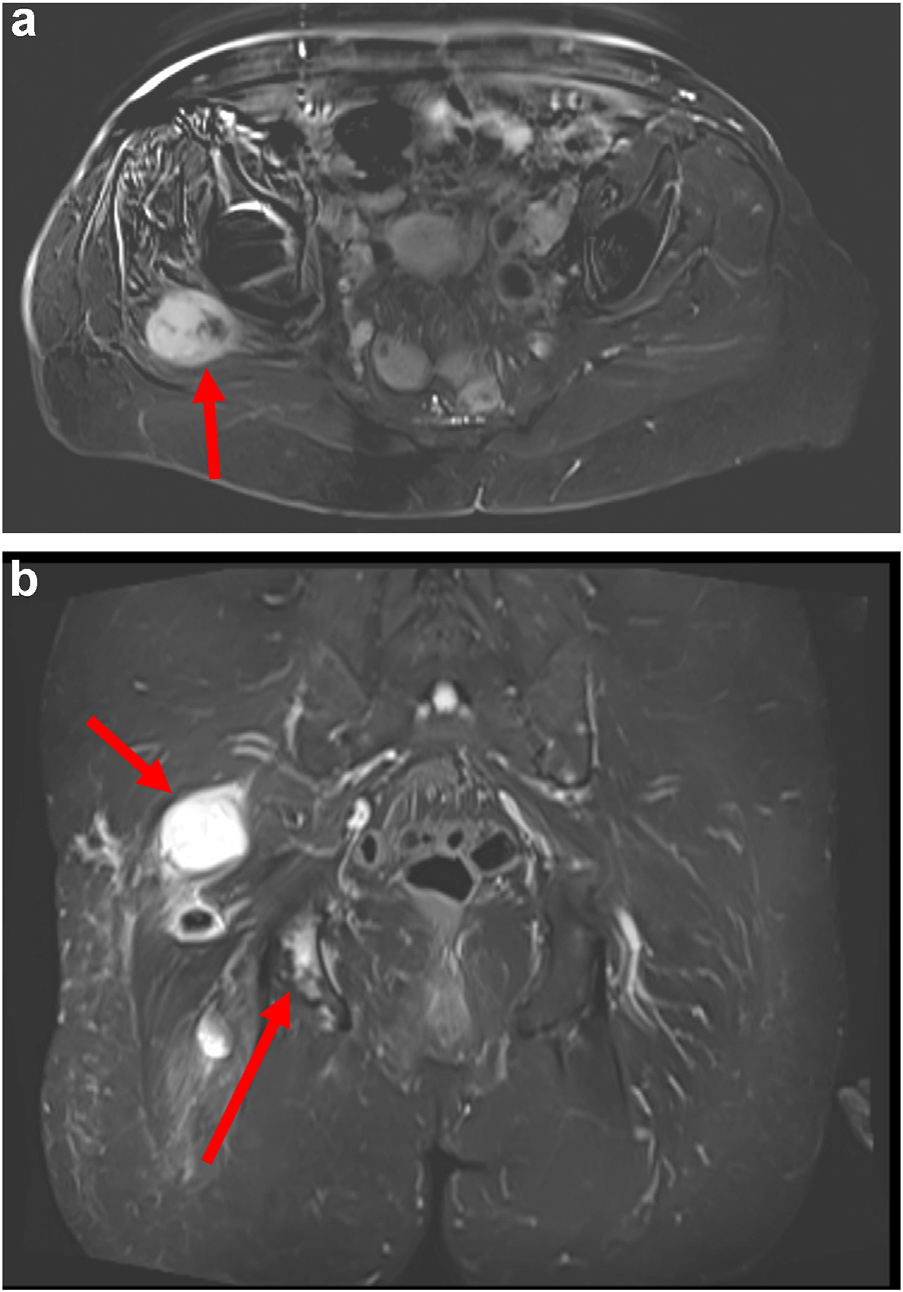
(a) Axial and (b) coronal T2 weighted MRI 5-year status postresection and proximal femoral replacement demonstrating hyperintense soft tissue mass posterior and superior to the right hip in the gluteal musculature as well as hyperintensity of the ipsilateral ischial tuberosity, which was later found to be a biopsy-proven recurrence of synovial sarcoma as well as associated osseous metastasis to the right ischium.

A short-interval scan showed further progression, and she was started on pazopanib, a tyrosine kinase inhibitor. Her lung metastases were stable for about 8 months but subsequently progressed. She started on a Phase 2 T-cell therapy clinical trial (NCT04044768). She had a partial response in her lungs but developed a local recurrence arising from the ischial tuberosity after 14 months on the trial. (See Fig. 7) She came off the trial, received radiation to the pelvis, and has had stable surveillance scans with no progression to date. At the latest follow-up, 6 and a half years from her initial SS resection, she ambulates with a walker/cane but has limited hip motion and strength due to extensive postradiation fibrosis.

## Discussion

Contrary to its name, SS is not typically found in the joints and has epithelial differentiation. They are characterized by a unique chromosomal translocation resulting in the formation of an oncogenic SS18-SSX fusion gene. [24] Locations can vary, but they are typically found in the soft tissues of the extremities, most commonly the thigh and knee [25]. Occurrence in extremities has been reported to be 68% in adults and 76% in children [26]. This tumor usually occurs in pediatric and young adult populations at a median age of 38.0 years. Only 11.9% of the cases occurred in patients >65 years old [27]. Males and females are affected equally, and symptoms can include a painful or painless mass with limited range of motion. [28] SS diagnosed in adults are often larger and have a higher rate of metastasis compared to those diagnosed in children. Tumor location can help predict the prognosis. Patients with extremity-based tumors have higher survival rates [28]. Overall, the 5-year survival rate was 62% in adults, which is lower than the 83% reported in children [26]. Treatment includes surgical excision followed by radiation and/or chemotherapy. It is important to note that the recurrence of SS happens later compared to other soft-tissue sarcomas after treatment. Most soft-tissue sarcomas recur in the first 2 years after treatment. In contrast, SS recurs at a mean of 3.6 years (range 0.5-15 years), and metastases occur at a mean of 5.7 years (range 0.5-16.3 years) [28].

The preponderance of relevant orthopaedic literature appears to lack sufficient data to demonstrate a widescale association between Co/Cr ion production and carcinogenesis. [[29], [30], [31], [32], [33], [34], [35]] However, despite contemporary clinical data on this topic, the in vivo effects of Co and Cr ion production emitted from THA prostheses remain poorly understood. In 2003, the International Agency for Research on Cancer concluded that Co was “possibly carcinogenic.” [36] In vitro studies demonstrate that Co ions cause direct DNA damage [14] and interfere with DNA repair. [13] The biologic reaction of Co/Cr ions are likely dependent on a multitude of undetectable and undeterminable factors such as ion concentration, anatomic location, and host genetic and immunologic factors. [37] Importantly, the mean follow-up times of many clinical studies evaluating an association between Co/Cr ions and malignancy may be shorter than the latency period of the malignancies they are attempting to observe. [[38], [39], [40], [41]] Interestingly, stratification of cancer sites has shown an increased incidence of cutaneous malignancies (melanoma and basal cell carcinoma) and hematologic malignancies (lymphoma and myeloma) in the presence of elevated Co/Cr ions. [38,39,41] It has been hypothesized that the increased risk of hematologic cancer is due to the exposure of hematopoietic cells to Co metal ions. [13,38,[42], [43], [44]]

Cobalt and chromium levels are not diagnostic of ALTR. A previous study had shown that patients with ALTR from MoM THA had Co and Cr levels of 2.25 μg/L (±3.98) and 2.39 μg/L (±3.41), while those in patients without ALTR had 0.88 μg/L (±0.64) and 1.80 μg/L (±1.23), respectively (*P* = .139 for Co and *P* = .504 for Cr) [45]. Patients with MoM THA and Co or Cr levels from 3.0-10.0 μg/L are considered at moderate risk of failure, and using a cutoff of ≥5 μg/L to diagnose ALTR provides a sensitivity and specificity of 63 and 86% [46]. Our patient with a Co level of 3.4 μg/L puts herself at moderate risk of failure.

We do not have the requisite evidence to definitively say this was a case of an ALTR that had transformed into a SS. However, certain facts involving this case should at least raise the hypothesis that this is the case. First, SS usually presents in individuals less than 40 years of age. Our patient was 69 years old. Secondly, the patient had elevated Cr-Co levels and MR evidence of a MoM THA associated mass. Thirdly, intraoperative findings included metallosis, likely caused by the particulate debris released from the bearing surface and mechanically-assisted crevice corrosion of the head-and-neck junction. Lastly, a soft tissue tract connected the mass to the hip joint. Given this data, we hypothesize that this was a case of an ALTR, a well-documented complication from MoM THA, which had transformed into SS.

## Summary

In light of our current report, the previous case report of a THA related telangiectatic sarcoma in 1988, [19] the aforementioned various malignancies, and considerable basic science data, we submit that a SS secondary to MoM THA should be a diagnostic possibility and not discounted based on current, limited clinical data. Considering the current inability to control for important factors such as ion concentration, rate of ion production, and host genetic, immunologic, and environmental factors, we pose that conclusions about this topic drawn from relevant clinical studies should be made cautiously. Nonetheless, when managing MoM ALTR and bearing failures, sarcoma should be included in the differential diagnosis, and the pursuit of further cytologic studies, including permanent section analysis and FISH, should be considered.

## Acknowledgments

The authors would like to acknowledge the Debartolo Center for Adult Reconstruction Research and Education, Depuy Synthes, and Tampa General Hospital for funding support for this research.

## Conflicts of interest

O. Binitie is a paid consultant for Onkos Surgical. B. T. Palumbo receives royalties/financial support, is a paid consultant, and is a speaker bureau of Enovis and Conformis; has stock options in Actuos Medical; and receives research support from Enovis. All other authors declare no potential conflicts of interest.

For full disclosure statements refer to https://doi.org/10.1016/j.artd.2024.101408.

## Informed patient consent

The author(s) confirm that written informed consent has been obtained from the involved patient(s) or if appropriate from the parent, guardian, power of attorney of the involved patient(s); and, they have given approval for this information to be published in this case report (series).

## CRediT authorship contribution statement

**Reed Andrews:** Writing – review & editing, Writing – original draft, Conceptualization. **Victor Shen:** Writing – review & editing, Writing – original draft. **Odion Binitie:** Writing – review & editing, Writing – original draft. **Brian T. Palumbo:** Writing – review & editing, Writing – original draft.

## Appendix A Supplementary Data

Conflict of Interest Statement for Binitie

Conflict of Interest Statement for Shen

Conflict of Interest Statement for Palumbo

Conflict of Interest Statement for Andrews
